# Sevoflurane preconditioning ameliorates neuronal deficits by inhibiting microglial MMP-9 expression after spinal cord ischemia/reperfusion in rats

**DOI:** 10.1186/s13041-014-0069-7

**Published:** 2014-09-04

**Authors:** Xiao-Qian Li, Xue-Zhao Cao, Jun Wang, Bo Fang, Wen-Fei Tan, Hong Ma

**Affiliations:** Department of Anesthesiology, First Affiliated Hospital, China Medical University, Shenyang, 110001 Liaoning China

**Keywords:** Apoptosis, Blood spinal cord barrier, Matrix metalloproteinase, Microglia, Neuron, Spinal cord ischemia/reperfusion injury

## Abstract

**Background:**

Microglia are the primary immune cells of the spinal cord that are activated in response to ischemia/reperfusion (IR) injury and release various neurotrophic and/or neurotoxic factors to determine neuronal survival. Among them, matrix metalloproteinase-9 (MMP-9), which cleaves various components of the extracellular matrix in the basal lamina and functions as part of the blood spinal cord barrier (BSCB), is considered important for regulating inflammatory responses and microenvironmental homeostasis of the BSCB in the pathology of ischemia. Sevoflurane has been reported to protect against neuronal apoptosis during cerebral IR. However, the effects of sevoflurane preconditioning on spinal cord IR injury remain unclear. In this study, we investigated the role of sevoflurane on potential genetic roles of microglial MMP-9 in tight junction protein breakdown, opening of the BSCB, and subsequent recruitment of microglia to apoptotic spinal cord neurons.

**Results:**

The results showed significant upregulation of MMP-9 in rats with IR-induced inflammation of the BSCB compared to that of the sham group, manifested as dysfunctional BSCB with increased Evans blue extravasation and reduced expression of occludin protein. Increased MMP-9 expression was also observed to facilitate invasion and migration of activated microglia, imaging as high Iba-1 expression, clustered to neurons in the injured spinal cord, as shown by double immunofluorescence, and increased proinflammatory chemokine production (CXCL10, CCL2). Further, sevoflurane preconditioning markedly improved motor function by ameliorating neuronal apoptosis, as shown by reduced TUNEL-positive cell counts and expression of cleaved caspase-3. These protective effects were probably responsible for downregulation of MMP-9 and maintenance of normal expression of occludin protein indicating BSCB integrity from inflammatory damage, which was confirmed by decreased protein levels of Iba-1 and MMP-9, as well as reduced production of proinflammatory chemokines (CXCL10, CCL2) and proinflammatory cytokines (IL-1β). Intrathecal injection of specific siRNAs targeting MMP-9 had similar protective effects to those of sevoflurane preconditioning.

**Conclusions:**

Preconditioning with 2.4% sevoflurane attenuated spinal cord IR injury by inhibiting recruitment of microglia and secretion of MMP-9; thus inhibiting downstream effects on inflammatory damage to BSCB integrity and neuronal apoptosis.

**Electronic supplementary material:**

The online version of this article (doi:10.1186/s13041-014-0069-7) contains supplementary material, which is available to authorized users.

## Introduction

Spinal cord ischemia/reperfusion (IR) injury is a well-recognized clinical problem resulting in motor and sensory dysfunction that is characterized as temporary or permanent ischemia of the spinal cord following reperfusion in a setting of shock or thoracoabdominal aorta surgery [1]. The blood brain barrier (BBB) is a well-known specialized structure that maintains brain tissue in an immune-privileged environment [1]. The spinal cord, as a part of the central nervous system (CNS), connects the brain and the peripheral nervous system. The blood-spinal cord barrier (BSCB) was presumed to represent both an anatomical and functional unit mediating molecular transport and immune regulation. The BSCB, consisting of a layer of tightly adhering endothelial cells lining the blood vessel lumen surrounded by astrocytes, pericytes and perivascular microglia, is critical for the barrier function by actively and selectively restricting the passage of water, ions, metabolites, and immune cells into the spinal cord under physiological and pathological states [2]. In a previous study using a rat IR model, we documented that increases in BSCB dysfunction and inflammatory reactions play an important role in the evolution of spinal cord IR injury and in the progression of neuronal damage [3,4]. Furthermore, accumulating evidence suggests that the extracellular matrix and tight junction complexes serve several important physiological functions, including defense and homeostatic balance between the spinal cord and systemic circulation, aberrant decomposition of which could cause neurotoxic effects [5,6]. Treatments that prevent decomposition of the extracellular matrix and tight junction complexes after IR may improve the prognosis of patients with spinal cord IR injury.

Matrix metalloproteinases (MMPs), a family of proteolytic enzymes, influence tissue injury and repair [7,8]. Among multiple nociception-related MMPs, levels of MMP-9, which is mainly secreted by glial cells, were markedly increased in cerebral IR models [7]. During the injury procedure, ischemia-induced oxidative stress and cytokines increased MMP-9 levels, which ultimately remodeled microvascular structures of the BBB and led to an influx of immune or inflammatory cells as well as subsequent secretion of chemokines and cytokines, thereby additionally stimulating both proliferation and recruitment of inflammatory cells into the CNS. Therefore, this self-sustaining vicious cycle of inflammatory damage to the BBB is tightly regulated by the expression of MMPs [8,9]. However, whether or how MMP-9 responds during spinal cord IR injury remains unknown.

Microglia, the primary immune effector cells of the spinal cord represent an important source of MMPs in the CNS [9]. They are situated very close to the BSCB to facilitate their robust activation in response to insult through a highly regulated network of cytokines and chemokines to subsequently orchestrate immune responses at sites of injury following BSCB opening [3,4,9,10]. Existing evidence shows that these extracellular matrix proteins could be a strong inducer of microglial activation [11], switching microglia from a resting state into an activated potentially destructive phenotype characterized by morphological changes, migration, proliferation, phagocytosis, antigen presentation, and secretion of diffusible factors including proinflammatory cytokines (such as TNF-α and IL-1β) [4,10,11]. Moderate activation of microglia is necessary for host defense, whereas over activation of microglia, together with excess secretion of proinflammatory chemokines and cytokines, is neurotoxic and promotes neuronal injury [3,10]. Recent papers report that neuronal cell death causes microglial activation by releasing various signaling molecules such as the chemokines, gamma interferon-inducible protein (CXCL) 10 and CC chemokine ligand (CCL) 2, as well as proinflammatory cytokines (e.g. IL-1β) and active forms of MMPs [9,12,13]. Thus, modulation of microglial activation and MMP-9 expression is considered to be a promising therapeutic strategy for IR injury. One control mechanism of MMPs is the interaction with TIMPs (tissue inhibitors of MMPs) [14]. Imbalances between MMPs and their inhibitors, resulting in excessive MMP proteolytic activity, are implicated in the pathogenesis of neuroinflammation [9,14]. Generally, all MMPs are inhibited by TIMPs once activated [14,15]. Therefore, we modulated MMP-9 expression by intrathecal injection of specific siRNAs targeting MMP-9 to further explore if IR-induced dysfunction is due to microglial MMP-9 [15].

Sevoflurane is the most widely used volatile anesthetic for both induction and maintenance of anesthesia in clinical practice. Mounting evidence indicates that sevoflurane preconditioning might induce ischemic tolerance in the brain and heart, and alleviate hypoxic and ischemic injury [16-18]. These findings have triggered an increasing interest in both basic science and clinical research. Previous studies demonstrate that sevoflurane can protect against ischemic neuronal damage and attenuate necrosis and apoptosis through its capacity to increase cerebral blood flow and preserve cerebral autoregulation [19]. However, few studies have attempted to describe the protective effect of this agent on spinal cord IR injury [16,20,21]. Therefore, the primary objective of this study was to investigate whether sevoflurane preconditioning can initiate protective effects on hind limb motor function and improve the viability of motor neurons in a rat model of transient aortic arch occlusion. Secondary objectives were to evaluate the correlation of microglial MMP-9 levels with downstream proinflammatory and apoptotic cytokines, such as CXCL10, CCL2, IL-1β, and caspase-3, with or without sevoflurane pretreatment.

## Results

All rats had normal neurological signs before the induction of ischemia and maintained good health throughout the experimental period.

### Sevoflurane preconditioning improved neurological assessment scores after IR injury

**Figure 1:**
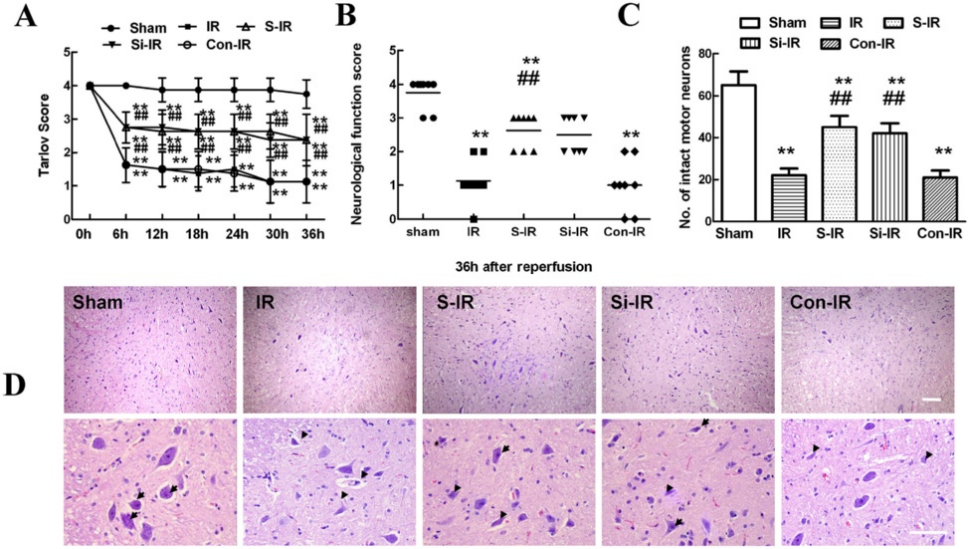
**Effects of sevoflurane preconditioning on neurologic motor function and histologic assessment of the spinal cord after ischemia/reperfusion (IR) injury. (A)** Neurological function scores were assessed at 6-h intervals during the 36 h observation using Tarlov scores after injury in three groups (n = 24). Neurological function scores ranged from 0 (paraplegia) to 4 (normal). Data are presented as the mean ± SEM. **(B)** Neurological function scores at 36 h after reperfusion in three groups. Each symbol represents data for one rat (n = 8, bar = median). **(C)** Number of intact motor neurons in the ventral gray matter (n = 8). **(D)** Representative sections of lumbar spinal cords in the ventral horn of gray matter stained with hematoxylin and eosin 36 h after reperfusion in three groups. Normal neurons exhibited a fine granular cytoplasm with Nissl substance (arrows), while dead neurons were identified by the presence of a diffuse cytoplasm without cellular structure and with extensive vacuolation of gray matter (arrowheads). Upper scale bar = 200 μm; lower scale bar = 100 μm. **P < 0.05 vs. sham group. ^##^ *P* < 0.05 vs. IR group.

Figure 1A shows the time course of neurological scores assessed by means of Tarlov scoring system ranged from 0 (paraplegia) to 4 (normal) during 36 h after reperfusion. There were significant decreases in average Tarlov scores (relative to baseline) in IR group (*P* <0.05), suggesting the development of motor function deficits induced by 14 min of thoracic aortic occlusion. And the average scores after IR were significantly improved in rats preconditioned with sevoflurane (*P* < 0.05). When evaluating the individual score in Figure 1B, each symbol represents for one rat. Compared with IR group, sevoflurane preconditioning statistically increased the number of rats with high scores and decreased the number of rats with low scores, suggesting sevoflurane preconditioning enhanced the recovery of neurological deficits (*P* < 0.05).

According to the results of our preliminary experiment that sevoflurane preconditioning’s rescue effects on IR-induced motor neuron dysfunction was probably through inhibition of MMP-9 upregulation (Additional file 1: Figure S1). So we also performed functional assays of MMP-9 with siRNA technology to further confirm the elevated levels of MMP-9 in IR procedure and explore whether reversing MMP-9 upregulation is one of the key elements for sevoflurane preconditioning. Similar to the effects of sevoflurane preconditioning, intrathecal pretreatment with MMP-9 siRNA was observed statistically higher average and individual Tarlov scores in comparison to those of IR group during 36 h after reperfusion (*P* < 0.05). There were no statistical differences between operated rats treated with or without control MMP-9 siRNA (*P* > 0.05).

### Sevoflurane preconditioning improved histologic assessment scores after IR injury

Histopathological changes 36 h after reperfusion are shown in Figure 1D. Compared that of sham controls, IR injury resulted in significant loss of motor neurons, as shown by the presence of diffusely eosinophilic cytoplasms without cell structure 36 h after surgery (*P* < 0.05), whereas more intact motor neurons with a fine granular cytoplasm and Nissl substance were found in operated-rats with sevoflurane preconditioning (sevoflurane preconditioning + IR group, S-IR group) (*P* < 0.05).

Similarly, downregulation of MMP-9 levels by intrathecal injection of MMP-9 siRNA had a similar neuroprotective effect to that of sevoflurane preconditioning, as shown by intact neurons comparable to those of the S-IR group (*P* > 0.05). Similarly, quantification data of intact neurons in spinal cord showed similar results in Figure 1C.

### Sevoflurane preconditioning improved neuronal apoptosis after IR injury

**Figure 2:**
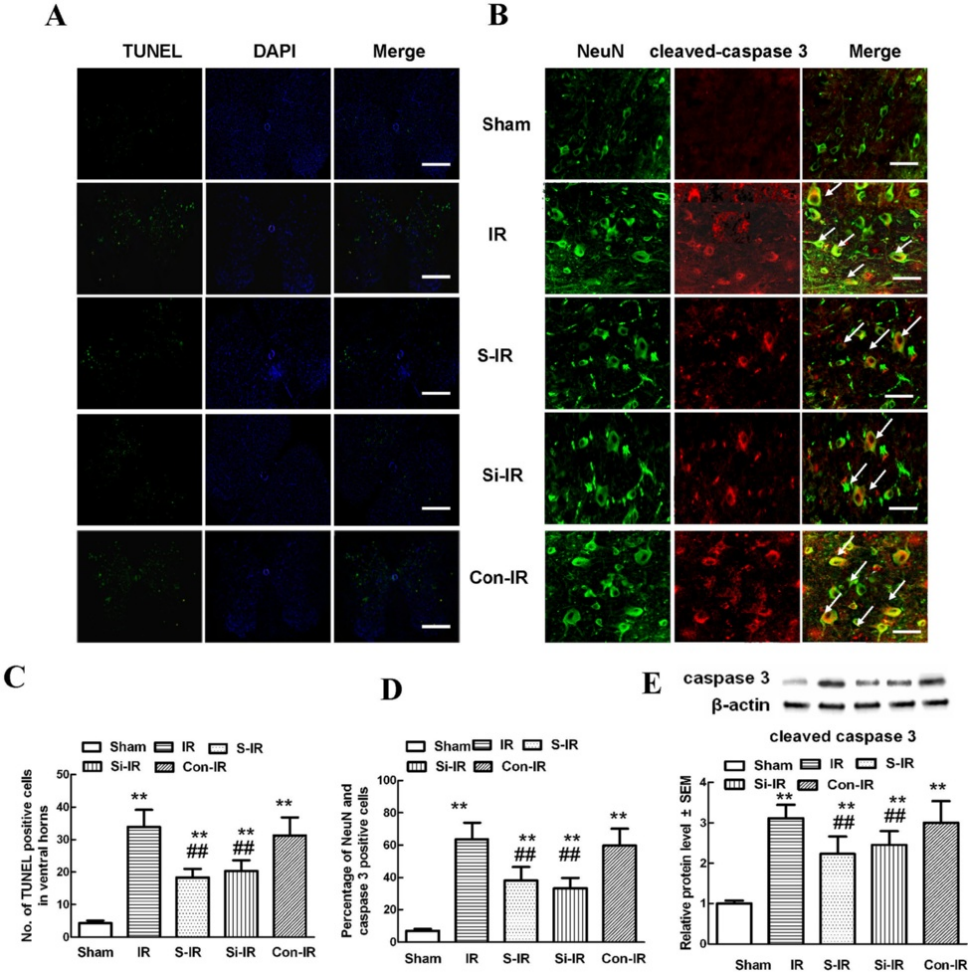
**Effects of sevoflurane preconditioning on neuronal apoptosis after spinal cord ischemia reperfusion (IR) injury. (A)** Representative micrographs of TUNEL staining (green) and DAPI (blue) in laminae IX of the ventral gray matter at 36 h after IR injury. Scale bars are 200 μm. **(B)** Representative immunohistochemical localization of neurons (NeuN; green) and cleaved-caspase-3 (red) in laminae IX of the spinal cord at 36 h after IR injury. Arrows delineate colocalization. Scale bars are 100 μm. Immunohistochemistry data showing that IR led to a decrease in neuronal number in the spinal ventral horn and an increased percentage of NeuN/cleaved-caspase-3-positive cells, suggesting the loss of neurons is partly a result of apoptosis. Pretreatment with sevoflurane and downregulation of MMP-9 by intrathecal injection of MMP-9 siRNA had neuroprotective effects reflected in decreased neuronal apoptosis. **(C)** Quantification of TUNEL-positive neurons in laminae IX of the ventral horn as averaged across three independent experiments. **(D)** Quantification of colocalized cells (cells with yellow signals) in laminae IX. **(E)** Representative western blot and quantitative protein analysis of cleaved caspase-3 in the spinal cord 36 h after surgery. Relative integral density values (IDVs) were calculated after normalizing to the sham group in each sample. All data are presented as mean ± SEM (n = 8 per group). **P < 0.05 vs. sham group; ^##^ *P* < 0.05 vs. IR group.

TUNEL staining is widely used to examine the integrity of DNA fragmentation as a marker of apoptosis [22]. As shown in Figure 2A, TUNEL assays showed that the fluorescent spots indicative of cell death mainly corresponded with the distribution of neuronal cell bodies. Quantitative analysis in Figure 2C showed that the amount of neuronal apoptosis in the IR group was significantly higher than that of the S-IR group and that apoptosis was very minimal in the sham group (*P* < 0.05). In addition, representative immunohistochemical analyses were performed to examine the expression of cleaved caspase-3, a known marker of apoptosis within neurons, and to investigate the cellular mechanisms by which treatments might attenuate the development of neuronal apoptosis [22]. Like the results of TUNEL staining, representative micrographs showed much stronger immunoreactivity of cleaved caspase-3 and more double-labeled neurons in IR group rats than in sham rats 36 h after injury, suggestive of neuronal apoptosis after IR. As expected, sevoflurane preconditioning reversed above changes (*P* < 0.05).

Similar to the effects of sevoflurane preconditioning, intrathecal pretreatment with MMP-9 siRNA is observed statistically weaker TUNEL staining and lower immunoreactivity of cleaved caspase 3 in neurons in comparison to those of IR group at 36 h after reperfusion (Figure 2B, *D*, *P* < 0.05). Finally, the same pattern was confirmed in cleaved caspase-3 protein levels in the spinal cord by western blot analysis (Figure 2E). There were no statistical differences between operated rats treated with or without control MMP-9 siRNA (*P* > 0.05).

### Sevoflurane preconditioning attenuated blood spinal cord barrier leakage

**Figure 3:**
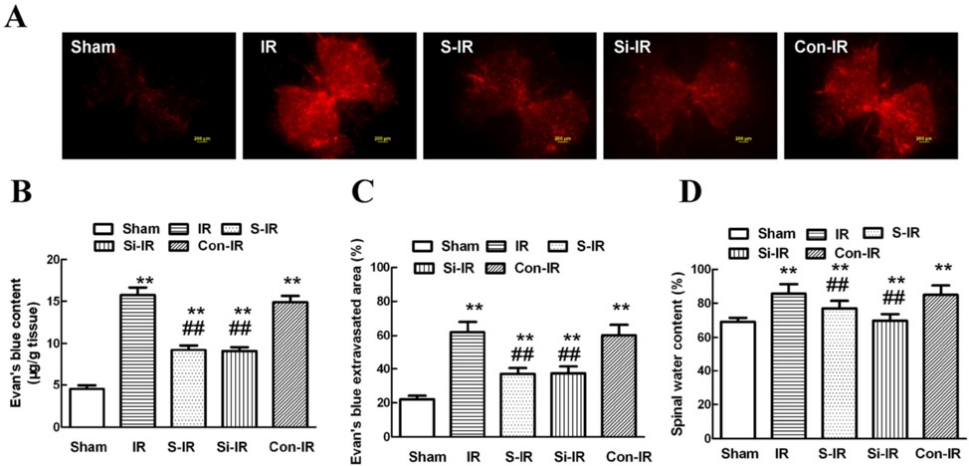
**Effects of sevoflurane preconditioning on blood-spinal cord barrier (BSCB) integrity after spinal cord ischemia reperfusion (IR) injury. (A)** Effects of spinal cord IR injury on BSCB permeability measured by EB extravasation. Almost no red fluorescence was observed in the spinal cord of sham group rats 36 h after injury. Much more red fluorescence, especially in the gray matter, was observed in IR group rats 36 h after injury, whereas EB red extravasation was significantly less in the sevoflurane preconditioning group. Further, downregulation of MMP-9 by intrathecal injection with MMP-9 siRNA had similar neuroprotective effects on the integrity of the BSCB to those of sevoflurane preconditioning. **(B)** EB content of the spinal cord (μg/g). **(C)** Percentage of EB extravasated area. **(D)** Quantification of the water content of the spinal cord (edema). All data are presented as mean ± SEM (n = 8 per group). Scale bars are 200 μm. **P < 0.05 vs. sham group; ^##^ *P* < 0.05 vs. IR group.

Extravasation of Evans blue (EB) is commonly used to evaluate the BSCB [9] and is visualized as red under a fluorescent microscope. As shown in Figure 3A, IR injury caused a marked increase in EB dye extravasation compared with that of the sham group 36 h after injury (*P* < 0.05); while sevoflurane preconditioning rescued the levels of extravasation following IR, indicating that sevoflurane preconditioning preserved BSCB integrity. Additionally, downregulation of MMP-9 levels by intrathecal injection of MMP-9 siRNA had comparable effects on reducing EB extravasation as those observed in the S-IR group, which was confirmed by quantitative analysis of EB content and extravasated area in Figure 3B, C (*P* > 0.05).

Besides, assessment of water content showed similar effects in Figure 3D because IR increased water content due to spinal cord edema closely related to damaged BSCB integrity. And there were no statistical differences between groups pretreated with sevoflurane or MMP-9 siRNA (*P* > 0.05), providing evidence that the neuroprotective effects of sevoflurane might result from MMP-9 downregulation.

### Sevoflurane preconditioning prevented microglia activation, migration, and upregulation of MMP-9 after IR injury

**Figure 4:**
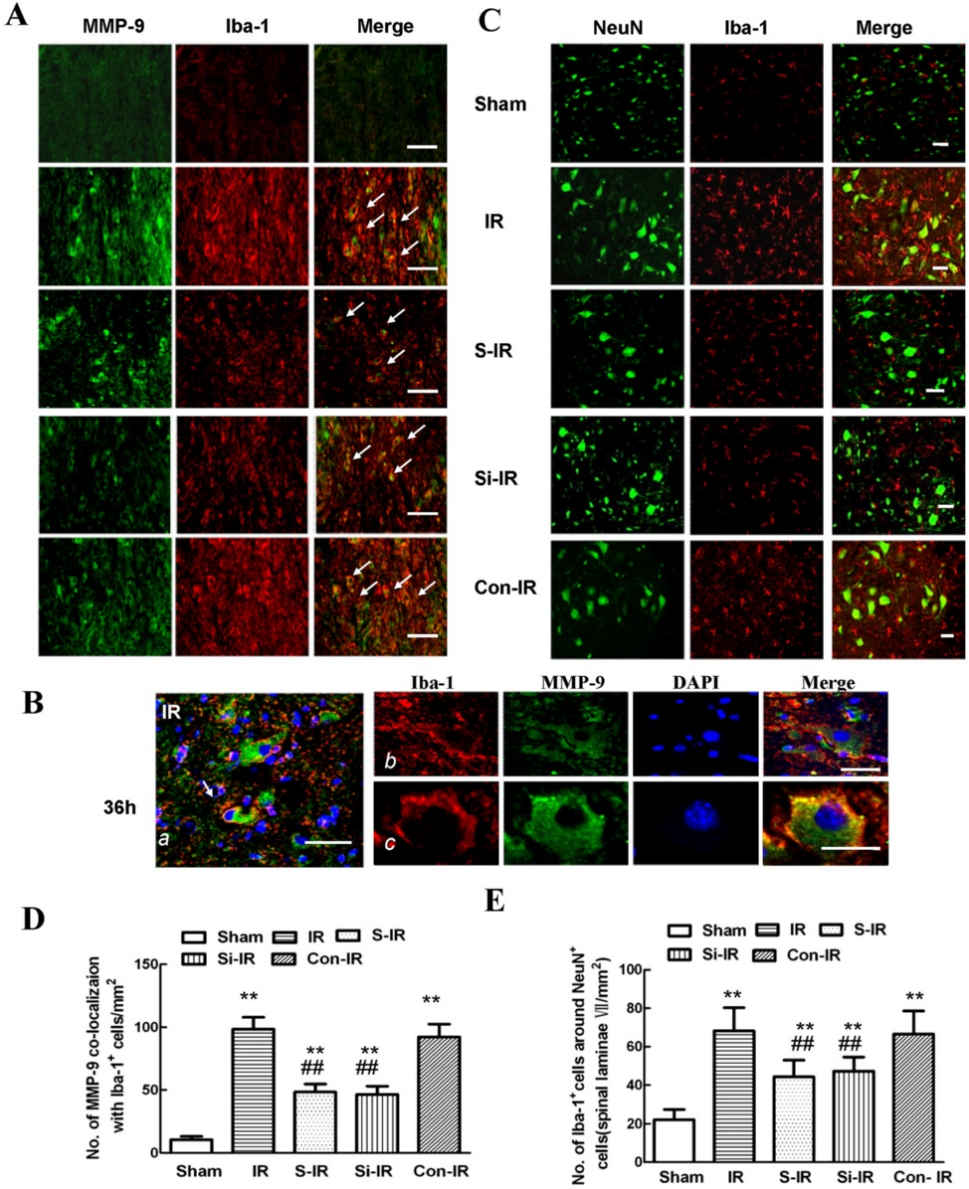
**Effects of sevoflurane preconditioning on microglia activity, migration, and secretion of MMP-9 after spinal cord ischemia reperfusion (IR) injury. (A)** Representative micrographs of the cellular location of MMP-9 (green) with antibodies against a microglial specific marker (Iba-1; red) 36 h after IR injury. Arrows delineate colocalization. A low magnification view showed fewer double-labeled cells in the spinal dorsal horn of rats preconditioned with sevoflurane or treated by intrathecal injection of MMP-9 siRNA. Arrows delineate colocalization. Scale bars are 100 μm. **(B)** Higher magnification image confirming that MMP-9 (with the identical fluorescence label) colocalized with Iba-1-positive cells 36 h after IR injury. Scale bars are 100 μm. **(C)** Distribution of Iba-1-positive cells in spinal lamina IX after IR injury. Representative micrographs showed that the IR-induced increase in Iba-1 immunoreactivity was prominently surrounded by NeuN-positive cells. Sevoflurane preconditioning prevented this redistribution. Similar neuroprotective effects were observed after downregulation of MMP-9 by intrathecal injection of MMP-9 siRNA. This suggests that the neuroprotective effects of sevoflurane preconditioning, in part, contributed to the inhibition of residual immune cell (microglia) activation and MMP-9 expression in the injured region. **(D)** Histogram for quantification of colocalized cells (cells with yellow signals). **(E)** Histogram for quantification of Iba-1-positive cells localized around NeuN-positive cells of lamina IX. All data are presented as mean ± SEM (n = 8 per group). **P < 0.05 vs. sham group; ^##^ *P* < 0.05 vs. IR group.

Microglia, the resident inflammatory and immune effectors in CNS, migrate into injured sites after insults [23]. To further confirm the relationship of neurons and activated microglial MMP-9 during IR injury, co-staining was performed using different protocols 36 h after injury with the following cell-specific markers: Iba-1 (microglial marker), NeuN (neuronal marker), and MMP-9. As shown in our previous study [3] and in Figure 4A,B, activated microglia with a macrophage-like morphology expressed high levels of Iba-1 36 h after IR injury. MMP-9 with the identical fluorescent label colocalized with the distribution of Iba-1-positive cells in the IR group. This was attenuated by preconditioning with sevoflurane or intrathecal injection of MMP-9 siRNA, although not in cells of the sham-operated animals (*P* < 0.05). Similar quantification of MMP-9 colocalization with Iba-1-positive cells (Figure 4D) confirmed that increased MMP-9 secreted by microglia was involved in IR-induced spinal cord injury.

Furthermore, when compared with those of the sham group, cells with robust Iba-1 immunoreactivity prominently surrounded neurons in the laminae of the spinal cord ventral horn 36 h post-IR injury; sevoflurane preconditioning inhibited the number of Iba-1-positive microglia and their migration to neurons (Figure 4C, 44.34 ± 8.73 versus 68.25 ± 12.06, *P* < 0.05). As expected, intrathecal treatment with MMP-9 siRNA had similarly inhibitory effects to those of sevoflurane preconditioning on the activation and migration of microglial cells (47.32 ± 7.34 versus 44.34 ± 8.73, *P* > 0.05), suggesting a close functional relationship between neuronal and microglial activation and increased MMP-9 expression in the injured region.

### Sevoflurane preconditioning inactivation of microglia results in reduced expression of MMP-9, proinflammatory chemokines and cytokines after IR injury

**Figure 5:**
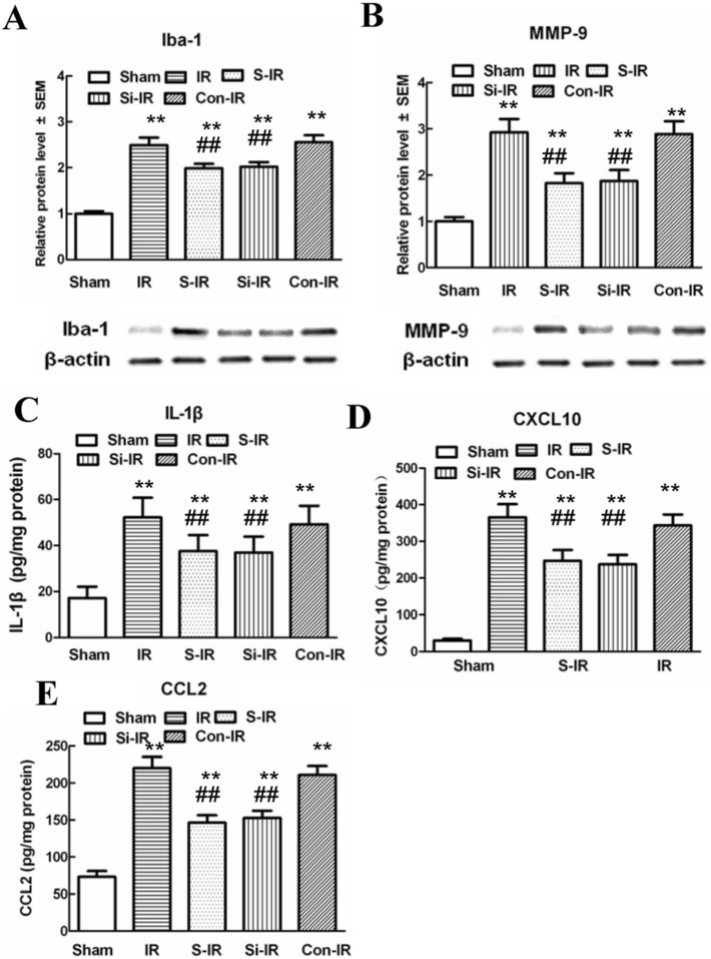
**Effects of sevoflurane preconditioning on proinflammatory chemokines and cytokines and neuronal apoptosis after spinal cord ischemia reperfusion (IR). (A-B)** Representative western blot and quantitative protein analysis of Iba-1 and MMP-9 in the spinal cord 36 h after surgery. Relative integral density values (IDVs) were calculated after normalizing to the sham group in each sample. **(C-E)** Quantification of CXCL-10, CCL2, and IL-1β production in the spinal cord 36 h after IR injury, as assessed by ELISA. Sevoflurane preconditioning prevented IR-induced microglial activation and significant increases in the products of proinflammatory chemokines (CXCL10 and CCL2) and cytokines (IL-1β) 36 h after IR injury. Similar anti-inflammatory effects were observed after intrathecal injection of MMP-9 siRNA. All data are presented as mean ± SEM. **P < 0.05 vs. sham group; ^##^P < 0.05 vs. IR group.

MMP-9 facilitates migration of microglia by disrupting the extracellular matrix and also modulates inflammatory reactions by cleaving inflammatory cytokines and chemokines [23,24]. To test whether sevoflurane preconditioning modulates the inflammatory response through regulation of microglial activation and secretion of MMP-9, we analyzed levels of Iba-1 and MMP-9 in injured spinal cords by western blot analysis and, in the case of CXCL10, CCL2, and IL-1β, by ELISA. Representative immunoblots and quantification (Figure 5A and B) showed that IR induced substantial increases in Iba-1 levels and secretion of MMP-9 in the spinal cord 36 h after IR surgery compared with those of sham-operated controls; whereas, significant decreases in the expression of Iba-1 and MMP-9 were detected in rats receiving sevoflurane preconditioning or intrathecal pretreatment with MMP-9 siRNA (*P* < 0.05). Meanwhile, spinal cord levels of proinflammatory chemokines (CXCL10 and CCL2) and cytokines (IL-1β) had similar expression profiles (Figure 5C-E, *P* < 0.05). There were no statistical differences between groups pretreated with sevoflurane or MMP-9 siRNA (*P* > 0.05), suggesting that sevoflurane preconditioning regulated the inflammatory response by reducing microglial activation and the secretion of MMP-9.

## Discussion

Physical damage to the spinal cord after ischemia/reperfusion (IR) injury is accompanied by increased blood spinal cord barrier (BSCB) permeability, followed by neuronal apoptosis, glial activation, and prolonged neuroinflammatory responses [6,14,25]. Considerable efforts have been directed at increasing ischemic tolerance in the spinal cord in both experimental animals and humans in order to decrease the incidence of paraplegia. Unfortunately, current therapies remain unsatisfactory and surgical management is still the main choice of treatment [4,6,26]. Sevoflurane, a volatile anesthetic with the unique clinical characteristics of rapid recovery time with little emergence agitation, is suitable for both induction and maintenance of anesthesia and as a result is the most frequently used anesthetic, clinically. Neuroprotective effects of sevoflurane preconditioning have been reported in several models of cerebral ischemia and are attributed to its capacity to increase cerebral blood flow for autoregulation [16,18,19]. Given the benefits of volatile anesthetic preconditioning in improving myocardial functional recovery and reduced infarct size after ischemia [17,27], we explored the therapeutic potential of treatment with sevoflurane before ischemic injury to induce a “preconditioned state” upon IR injury. Consistent with previous research in our laboratory [16,19], the present study demonstrated that spinal cord IR injury induced by 14-min aortic arch occlusion produced extensive neuronal apoptosis manifesting as severe hind-limb motor functional deficits, along with increased BSCB leakage. Sevoflurane preconditioning 1 h prior to ischemia led to significant improvements in BSCB integrity and motor function due to decreased microglial activation resulting in reduced expression of MMP-9, proinflammatory chemokines (CXCL10 and CCL2), and cytokines (IL-1β) after IR injury. In parallel with the role of sevoflurane preconditioning in the course of IR, we found similar profiles in rats intrathecally pretreated with siRNA, suggesting that sevoflurane regulates MMP-9-evoked pronociceptive actions after IR. These results confirm and extend previous studies of preconditioning induced by volatile anesthetic agents [17-20].

Microglial cells are key responders to neural infarcts and play a central role in regulating the pathogenesis of IR injury [3,4,10,28]. As the resident macrophages of the spinal cord, they retract and thicken their cellular processes to transform into an amoeboid form within 30 min of neural insult and migrate toward vulnerable areas to confine the lesion and are capable of phagocytosis [3,7,11,28]. However, some studies suggest that microglia activation has both positive and negative effects in various neurological models and conditions [28]. In addition to releasing neurotrophic factors, activated microglial cells also secrete various pro-inflammatory cytokines, nitric oxide, and other factors that can aggravate injury [3,11,28,29]. How the specific timing and extent of microglial activation might affect these deleterious versus beneficial consequences for spinal cord recovery remains a controversial topic. Karwacki Z and colleagues reported that sevoflurane in concentrations of 1 and 2 MAC (minimum alveolar concentration) inhibited ischemia-induced polymorphonuclear neutrophil (PMN) adhesion. With similar immunophenotypical features as those of PMN, sevoflurane was demonstrated to modulate microglial adhesion [30]. Herein, we used Iba-1 as cellular marker of microglia and analyzed microglial activation qualitatively and quantitatively. Our data show that after IR injury, microglia were rapidly activated in IR group rats, as cells were intensely stained with Iba-1 antibody and possessed enlarged and vacuolated cell bodies extending stout processes (Figure 4A). Sevoflurane preconditioning markedly prevented increases in Iba-1 protein expression (Figure 5A), completely consistent with the results of Karwacki Z and colleagues [30].

In case of IR, infiltration and migration of microglia into the spinal cord have been linked with injury to the neurovasculature caused by substances released from activated microglia, namely matrix metalloproteinases (MMPs) [7,14]. MMPs are capable of degrading extracellular matrix components and are responsible for promotion of BSCB opening [9,14,30]. Among MMPs, MMP-9 has been reported to be the most abundant inducible factor in the pathology of ischemia in several previous studies [8,9,11,31] including our rabbit IR model [6]. In the present study, we also found that fluorescently labeled MMP-9 colocalized with Iba-1-positive cells in the injured spinal cords of rats subjected to IR injury, and sevoflurane preconditioning prevented the increased number of such double-labeled microglia (Figure 4A). Correspondingly, western blot analysis (Figure 5A) confirmed the results of the immunofluorescence analysis. Representative immunoblots showed that reduced MMP-9 protein expression was associated with low expression of Iba-1, 36 h after surgery. These results were consistent with the study of Matsmumoto and colleagues [32], who also found that the initial changes in MMP activity could be detected as early as 15 min after insult and persisted for up to 48 h; a time frame that exactly coincides with the morphological and functional changes of microglia [12,32]. As expected, in the present study, intrathecal infusion of MMP-9 siRNA had a suppressive effect on the number of double-labeled activated microglia and on MMP-9 activity. In addition to extracellular remodeling of the BSCB, MMP-9 has anti-apoptotic and anti-inflammatory effects in various models [8,9,32-34]. It is well known that apoptosis is one of the major mechanisms that leads to cell death after IR. After ischemia, DNA fragmentation and chromatin condensation function as pro-apoptotic signals that trigger caspase activation [22,25,26]. The present study also demonstrated that sevoflurane preconditioning influences motor function by likely inhibiting infiltration and migration of microglia to injured neurons, in accordance with the phenomena described by Choi MS and colleagues that microglia, the resident effectors of the CNS, migrate into injured sites to initiate neuronal inflammation after insults [23]. Moreover, we performed TUNEL staining and measured protein levels of cleaved caspase-3, a known marker of apoptosis within neurons, to investigate the mechanisms by which sevoflurane might attenuate the development of neuronal apoptosis after IR (Figure 2A, B). The data showed that TUNEL-positive cells mainly displayed a neuronal morphology and were distributed in the lamina of the anterior horn with increased cleaved caspase-3 immunoreactivity, which were further confirmed by similar changes in caspase-3, Iba-1, and MMP-9 protein levels observed in western blots. Furthermore, downregulation of MMP-9 expression by intrathecal pretreatment with siRNA had similar anti-apoptotic effects as those of sevoflurane preconditioning, suggesting that microglia produce the majority of MMP-9 in response to IR injury, ultimately leading to neuronal apoptosis and motor function impairments.

In regard to inflammation, MMP-9 facilitates migration of inflammatory cells by cleaving inflammatory cytokines and chemokines in several inflammatory diseases, such as Alzheimer's disease, Parkinson disease, and multiple sclerosis, or after stroke or spinal cord trauma [23]. CXCL10 and CCL2 are involved in blood cell and PMN chemotaxis [13,35–37]. Moreover, a few studies also have reported that CXCL10 and CCL2 were chemokines that acted as potent recruiters with their primary effects on the innate immune system via chemotaxis residual microglia during peripheral and CNS inflammation [35,36,38]. Our study suggests that IR-induced upregulation of MMP-9 expression drives production of proinflammatory chemokines (CXCL10 and CCL2) and cytokines (IL-1β) that in turn amplify inflammatory responses, which ultimately promote neuronal apoptosis leading to hind-limb motor dysfunction [23,37]. Thus, decreased expression could contribute to reduced infiltration or migration of resident microglia of the spinal cord, leukocytes, and other proinflammatory cells in circulation through the disrupted BSCB, thereby ameliorating the inflammatory response [35-37,39]. Notably, prophylactic administration of neutralizing antibodies against CXCL10 and CCL2 prevents microglial infiltration, decreases neuronal loss, and improves behavioral recovery from spinal cord injury [37]. Further, in support of the above hypothesis, intrathecal pretreatment with specific siRNAs targeting MMP-9 had similar effects on reducing the release of chemokines (CXCL10 and CCL2) and products of proinflammatory cytokines ( IL-1β) leading to decreased neuronal loss and improved behavioral recovery from spinal cord injury (Figures 2 and 4). This inhibition by MMP-9 siRNA may limit the extent of BSCB disruption as well as the extent of microglial activation and progressive recruitment to the spinal cord, which correlated with caspase 3 expression and inflammation-mediated neuronal loss [15]. One the basis of published work, IL-1β is well-recognized as proinflammatory proteins that are associated with activated microglia [3,4,35]. Meanwhile, IL-1β is activated via cleavage from its precursor by caspase-1 (the IL-1β converting enzyme), as well as by other enzymes such as MMP-9 and MMP-2 [15]. Consistent with previous studies, the data in Figure 5 also show that IL-1β expression was increased in the spinal cord in a microglial activity-dependent manner during the course of IR [4,10]. Intrathecal MMP-9 siRNA treatment abrogated the increase in IR-induced IL-1β content, suggesting that IL-1β activation was a downstream mechanism underlying MMP-9-induced neuroinflammation accompanied by microglial activation [13]. Thus, it is conceivable that MMP-9 might antagonize these inflammatory factors via inhibiting the release of chemokines (CXCL10 and CCL2) and proinflammatory cytokines (IL-1β), which is in agreement with recent studies by Kjell J and Huang CY [38,40]. They further suggested that neurons and microglia form a highly interdependent network that is now considered to be the functional unit of the CNS. Although it is unknown whether microglia are the first responders to injury or respond to an “emergency signal” from neurons, it is conceivable that neuronal apoptosis following IR injury combined with extracellular microenvironmental imbalances might be the major stimuli for microglial activation [4,28,31]. All the above evidence, when considered together, suggests that upon IR-induced activation, microglia express increased CXCL10, CCL2, and other chemotaxis-associated molecules, which leads to the recruitment of inflammatory cytokines to the lesional area, resulting in amplified activation of additional spinal cord-derived microglia leading to neuronal loss. Blocking this pathological sequence by sevoflurane preconditioning had beneficial effects on BSCB integrity and hind-limb functional recovery. Nevertheless, it's worth noting that that the neuroprotective effects of sevoflurane preconditioning against spinal ischemia is very complicated. Some other underlying mechanisms such as activation of extracellular signal-regulated kinase or potassium ATP channel, as well as through free radicals-mediated up-regulation of antioxidant enzymes were also demonstrated to play a role [16-18,26,41]. Further studies focused on the links between different actions of sevoflurane preconditioning still need to be conducted.

In conclusion, the current study provides the first direct evidence that sevoflurane preconditioning produces anti-inflammatory and anti-apoptotic effects on spinal cord IR injury in rats. We provide additional evidence that this effect is possibly mediated through the inactivation of microglia resulting in reduced expression of MMP-9 and proinflammatory chemokines and cytokines. These data suggest that sevoflurane preconditioning may provide a new practical method for protecting against perioperative spinal cord IR injury.

## Materials and methods

### Experimental animals and protocols

The study was performed in accordance with the Guide for the Care and Use of Laboratory Animals (U.S. National Institutes of Health publication No. 85–23, National Academy Press, Washington DC, revised 1996). The experimental protocol was approved by the Animal Care and Use Committee of China Medical University. Male Sprague–Dawley rats, weighing between 200 and 250 g, and neurologically intact before anesthesia were used in our experiment. Rats were acclimatized for at least 7 days prior to the operation and were bred in standard cages on a 12 h light/dark cycle with free access to food and water. Seventy-two male SD rats were assigned by means of a random number table to receive either 33% oxygen or sevoflurane preconditioning (33% oxygen + 1 MAC sevoflurane) for 1 h, followed by a 30-min washout. The sham group rats were subjected to sham operations without IR injury. The IR group rats were subjected to a 14-min occlusion of the aortic arch followed by reperfusion. Sevoflurane preconditioning + IR group rats (S-IR) were pretreated with inhalation of sevoflurane before induction of IR injury. In parallel, IR + MMP-9 siRNAs or control RNA group rats (Si-IR or Con-IR) were intrathecally injected with 5 μg MMP-9 siRNA or control RNA (Jima Inc., Shanghai, China) at a concentration of 1 μg /μL once a day starting 2 days before ischemia. The sequences of these siRNAs were as follows. MMP-9 siRNA: 5′-GACUUGCCGCGAGACAUGAtt-3′ (forward), 5′-UCAUGUCUCGCGGCAAGUCtt-3′ (reverse); control RNA: 5′-GACUUCGCGGGACACAUGAtt-3′ (forward), 5′-UCAUGUGUCCCGCGAAGUCtt-3′ (reverse) [15].

### Sevoflurane preconditioning and surgical procedures

Preconditioning with sevoflurane was performed as previously described [17]. In brief, rats were exposed to 2.4% sevoflurane in a 33% oxygen and air mixture by breathing spontaneously through a nonsealing facemask device. The end-tidal sevoflurane concentration in expired gas was continuously monitored and maintained at 1 MAC (2.4–2.7%) with a gas analyzer (Capnomac Ultima; Datex, Helsinki, Finland) for 1 h. In all groups, rectal temperature was measured and maintained at 37.0 ± 0.5°C with a heated operating table. Rats were anesthetized by intraperitoneal injection of 4% sodium pentobarbital at an initial dose of 50 mg/kg after 2 h. The spinal cord IR model was induced by occlusion of the aortic arch for 14 min, as previously reported [42]. The aortic arch was exposed through a cervicothoracic approach. Under direct visualization, the aortic arch was cross-clamped between the left common carotid artery and the left subclavian artery. A catheter was inserted into the left carotid artery and into the tail artery to measure proximal and distal blood pressure. Ischemia was confirmed as a 90% decrease in flow measured at the tail artery using a laser Doppler blood flow monitor (Moor Instruments, Axminster, Devon, UK) for 14 min, after which, the clamp was removed and reperfusion was performed for 36 h. Sham operation rats underwent the same procedure, but no occlusion of the aortic arch was performed. All rats were allowed to recover in a plastic box at 28°C for 4 h and were subsequently placed in separate cages with free access to food and water. Rats were euthanized 36 h after the surgical procedure.

### Neurologic assessment

After reperfusion, neurologic function was quantified by two observers who were blinded to the experimental procedures at a 6 h interval during a 36 h observation period using a Tarlov scoring system, as follows: 0 = no lower limb function; 1 = perceptible lower limb function with weak antigravity movement only; 2 = some movement of lower limb joints with good antigravity strength, but inability to stand; 3 = ability to stand and walk, but not normally; and 4 = normal motor function [40].

### Hematoxylin and eosin staining

All rats were anesthetized with an overdose of pentobarbital 36 h after IR injury and the L4–L6 segments of spinal cords were rapidly collected for analysis because of their vulnerability to ischemic injury. Tissue was fixed in 4% paraformaldehyde and embedded for hematoxylin and eosin (HE) staining. Stained sections were examined under a microscope by two observers unaware of the grouping and outcomes (Olympus reflected and visual communication system, Olympus, Japan) to judge the morphological appearance of neurons. Normal neurons were defined as viable by the presence of basophilic stippling (containing Nissl substance), whereas necrotic or dead neurons were identified by the presence of a diffusely eosinophilic cytoplasm with pyknotic homogenous nuclei, according to our previously validated protocol [16]. Data were calculated as the mean number of intact neurons per area in three sections for each rat.

### Blood spinal cord barrier leakage evaluation

At 12 h and 36 h after IR injury, EB content and EB fluorescence were used for quantitative and qualitative examination of BSCB integrity, as previously described [9,12]. Briefly, 30 g/L (45 mg/kg; Sigma) EB was slowly administered in the tail vein 60 min before sacrificing the animals. After soaking the tissues in methanamide for 36 h (60°C) and centrifugation, the absorption of the supernatant was detected at 632 nm and reported as the amount of EB per wet tissue weight (μg/g) to quantify extravasation. In addition, the tissue was fixed in 4% paraformaldehyde (PFA), then sectioned (10 μm) and visualized using a BX-60 (Olympus, Melville, NY) fluorescence microscope (green zone) for fluorescence measurements.

### Double immunofluorescence

To measure the expression of MMP-9 in microglia, double immunofluorescence staining of MMP-9 and the microglia marker Iba-1 was performed as described previously [3,4,11]. Briefly, the spinal cord was fixed and sectioned into 10-μm slices with a Leica CM3050 S cryostat. Sections were blocked with 10% bovine serum albumin (BSA) for 1 h at room temperature and incubated overnight at 4°C with primary antibodies: rabbit anti-Iba-1 antibody (1:800, Wako, 019–19741) together with mouse anti-MMP-9 (1:200, Abcam 58803) or mouse anti-neuronal specific nuclear protein (NeuN, 1:500; Abcam 104224). After incubation for 2 h at room temperature with the corresponding secondary antibodies: Alexa 488-conjugated donkey anti-rabbit IgG (1:500, Molecular Probes, Rockford, USA) and Alexa 594-conjugated donkey anti-mouse IgG (1:500, Molecular Probes), sections were incubated with 4′,6-diamidino-2-phenylindole (DAPI; 1:500000; Abcam 104139) for 10 min at room temperature before cover slipping. Sections were examined and imaged with a Leica TCS SP2 (Leica Microsystems, Buffalo Grove, IL, USA) laser scanning spectral confocal microscope.

### TUNEL assay

A TUNEL (terminal deoxynucleotidyl-transferase-mediated dUTP nick-end labeling) assay is the most commonly used technique for examining apoptosis via DNA fragmentation. In situ detection of apoptosis in spinal cords was performed through staining using an ApopTag® Fluorescein In Situ Apoptosis Detection Kit (EMDMillipore, S7110) to modify genomic DNA utilizing terminal deoxynucleotidyl transferase (TdT), according to the manufacturer’s instructions. After TUNEL labeling, cell nuclei were labeled with DAPI (1:500000; Abcam 104139), and examined under a laser scanning spectral confocal microscope by two independent observers. Average numbers of TUNEL-positive motor neurons in the anterior spinal cord of the three sections were counted for comparisons among the groups.

### Western blot analysis

Expression of MMP-9 and NF-κB in spinal cord tissue was determined by western blot analyses. Rat spinal cords were homogenized and total proteins were purified using cell and tissue protein extraction reagents according to the manufacturer’s instructions (KC-415; Kang Chen, Shanghai, China). The antibodies used for western blotting were mouse monoclonal anti-Iba-1(1:800, Abcam 15690), rabbit polyclonal anti-MMP-9 (1:500, Abcam 38898), and rabbit monoclonal anti-cleaved caspase-3 (1:500, Abcam32351), with horseradish peroxidase-conjugated secondary antibodies (Bioss, Beijing, China) for the corresponding species. Blots were also probed with rabbit monoclonal anti-GAPDH (1:1000; Abcam 8245) as a loading control.

### ELISA analysis

Spinal cords were collected, homogenized, and centrifuged to obtain tissue for ELISA. Chemokine (CXCL10 and CCL2) and proinflammatory cytokine (IL-1β) content were determined using ELISA kits (Pharmingen, San Diego, Calif; R&D Systems, Minneapolis, MN, US). According to the manufacturers’ instructions, absorbance (A) was quantified at λ = 450 nm. The IL-1β content of each sample was calculated based on a standard curve. CXCL10, CCL2, and IL-1β concentrations were expressed in pg/mg protein.

### Statistical analysis

All data were expressed as means ± standard error of the mean (means ± SEM) and analyzed by SPSS software (version 17.0, SPSS Inc, Chicago, IL, USA). All variables measured in this study were normally distributed. Groups were compared with Student’s *t*-test or one-way analysis of variance (ANOVA), followed by Newman-Keuls post-hoc analysis. A *P* value of <0.05 was considered statistically significant.

## Additional file

Additional file 1: Figure S1. Effects of sevoflurane preconditioning on MMP-9 expression in the spinal cord after ischemia reperfusion (IR). (A) Representative western blot and quantitative protein analysis of MMP-9 in the spinal cord 36 h after surgery. Relative IDVs were calculated after normalizing to the sham group in each sample. (B) Real-time PCR analysis was performed in duplicate for MMP-9 under study. All data are presented as mean ± SEM. **P < 0.05 vs. sham group; ^##^P < 0.05 vs. IR group.

## Abbreviations

BBB: Blood brain barrier
BSCB: Blood-spinal cord barrier
CCL2: CCL (C-C motif ligand) 2
CXCL10: Gamma interferon-inducible protein 10
CNS: Central nervous system
DAPI: 4′,6-diamidino-2-phenylindole
EB: Evans blue
Iba-1: Ionized calcium-binding adaptor molecule 1
IL: Interleukin
IR: Ischemia/reperfusion
MAC: Minimum alveolar concentration
MMP: Matrix metalloproteinase
NeuN: Neuronal nuclei
PMN: Polymorphonuclear neutrophil
TUNEL: Terminal deoxynucleotidyl-transferase-mediated dUTP nick-end labeling
