# Supplementary figures and images for: Sevoflurane preconditioning ameliorates neuronal deficits by inhibiting microglial MMP-9 expression after spinal cord ischemia/reperfusion in rats
Source: Mol Brain. 2014 Sep 4;7:69. doi: 10.1186/s13041-014-0069-7 (PMC4161899; doi:10.1186/s13041-014-0069-7)

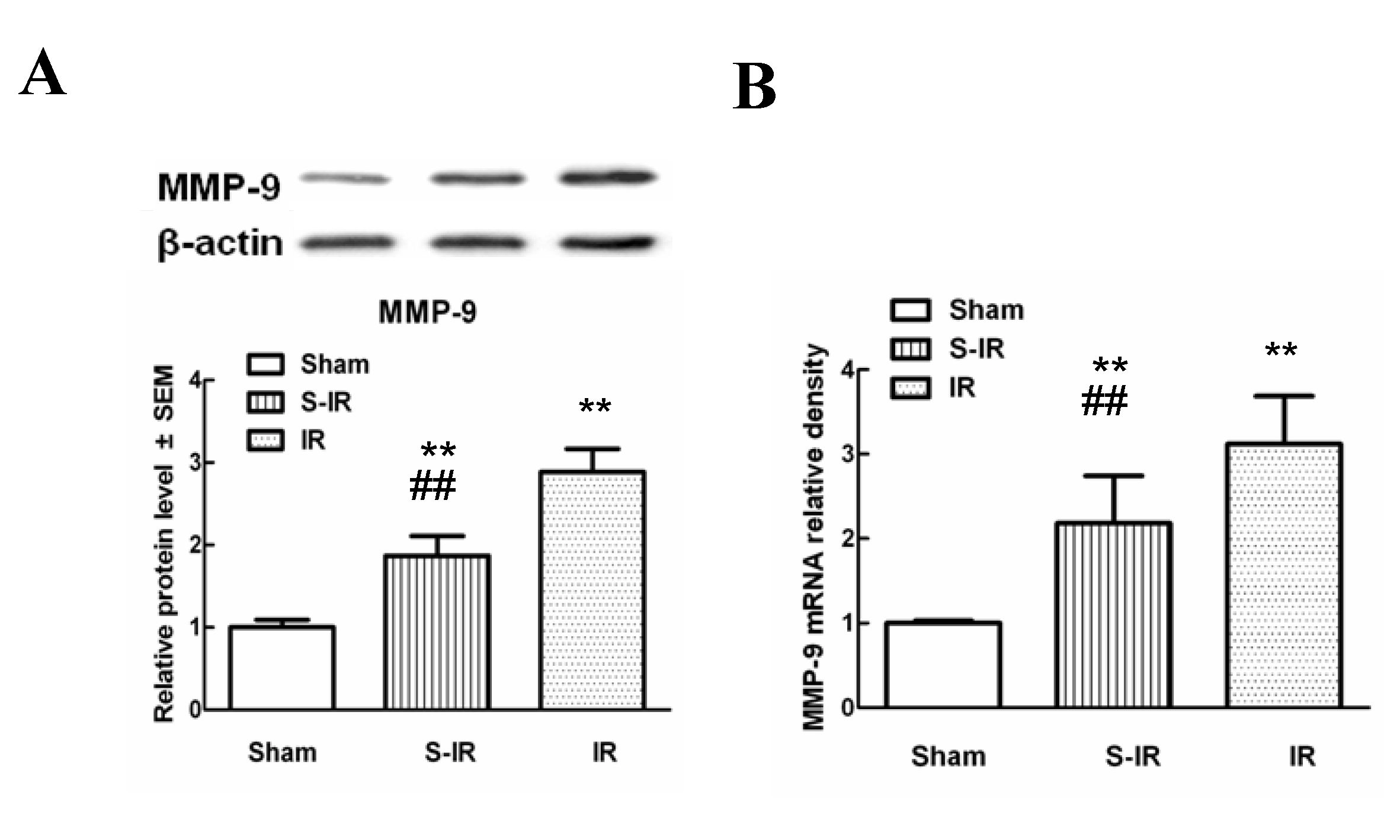

Supplement: Additional file 1: Figure S1. — Effects of sevoflurane preconditioning on MMP-9 expression in the spinal cord after ischemia reperfusion (IR). (A) Representative western blot and quantitative protein analysis of MMP-9 in the spinal cord 36 h after surgery. Relative IDVs were calculated after normalizing to the sham group in each sample. (B) Real-time PCR analysis was performed in duplicate for MMP-9 under study. All data are presented as mean ± SEM. **P < 0.05 vs. sham group; ##P < 0.05 vs. IR group. [file 13041_2014_69_MOESM1_ESM.tiff]
